# Perspectives of Major World Religions regarding Euthanasia and Assisted Suicide: A Comparative Analysis

**DOI:** 10.1007/s10943-022-01498-5

**Published:** 2022-01-29

**Authors:** Graham Grove, Melanie Lovell, Megan Best

**Affiliations:** 1 QLD Specialist Palliative Rural Telehealth Service, Robina Hospital, Robina, QLD 4226 Australia; 2 Bond University, Robina, QLD 4226 Australia; 3 School of Medicine, Griffith University, Southport, QLD 4222 Australia; 4 University of Sydney, Camperdown, NSW 2050 Australia; 5 Renew Church Gold Coast, Gold Coast, QLD 4213 Australia; 6 HammondCare Palliative Care Services, Greenwich, NSW 2065 Australia; 7 University of Sydney, Camperdown, NSW 2050 Australia; 8 Institute for Ethics and Society, University of Notre Dame, Chippendale, NSW 2007 Australia

**Keywords:** Euthanasia, Assisted suicide, Religion

## Abstract

Euthanasia and physician-assisted suicide (EPAS) are important contemporary societal issues and religious faiths offer valuable insights into any discussion on this topic. This paper explores perspectives on EPAS of the four major world religions, Christianity, Islam, Hinduism and Buddhism, through analysis of their primary texts. A literature search of the American Theological Library Association database revealed 41 relevant secondary texts from which pertinent primary texts were extracted and exegeted. These texts demonstrate an opposition to EPAS based on themes common to all four religions: an external locus of morality and the personal hope for a better future after death that transcends current suffering. Given that these religions play a significant role in the lives of billions of adherents worldwide, it is important that lawmakers consider these views along with conscientious objection in jurisdictions where legal EPAS occurs. This will not only allow healthcare professionals and institutions opposed to EPAS to avoid engagement, but also provide options for members of the public who prefer an EPAS-free treatment environment.

## Introduction

Euthanasia and physician-assisted suicide (EPAS) is an important issue in contemporary societies, increasingly discussed in medical, legal and religious organisations. The topic often elicits strong opinions, with some in favour and others against the legalisation of EPAS. Several jurisdictions have legalised EPAS in one form or another in the last three decades, including the Netherlands, Belgium, Switzerland, Canada, Spain and various states of the USA and Australia (Table 1).

**Table 1:** Countries with some form of legal EPAS and their religious make-up

Region where some form EPAS is legal	Religious and secular features	
Worldwide average	Not religious or affiliated with a religion	16%
	Affiliated with a religion	84%
	Christian 32%	
	Muslim 23%	
	Hindu—15%	
	Buddhist—7%	
	Other—9%	
	Religion considered very important	54%
Belgium	Not religious or affiliated with a religion	29%
	Affiliated with a religion	70%
	Christian: 64%	
	Other: 6%	
	Religion considered very important	11%
California, USA	Not religious or affiliated with a religion	28%
	Affiliated with a religion	73%
	Christian—63%	
	Other—10%	
Canada	Not religious or affiliated with a religion	24%
	Affiliated with a religion	76%
	Christian—69%	
	Other—7%	
	Religion considered very important	27%
Colombia	Not religious or affiliated with a religion	7%
	Affiliated with a religion	
	Christian—92%	
	Other—1%	93%
	Religion considered very important	77%
Colorado, USA	Not religious or affiliated with a religion	31%
	Affiliated with a religion	69%
	Christian—64%	
	Other—5%	
Hawaii, USA	Not religious or affiliated with a religion	27%
	Affiliated with a religion	73%
	Christian—63%	
	Other—10%	
Luxembourg	Not religious or affiliated with a religion	27%
	Affiliated with a religion	73%
	Christianity—71%	
	Other—2%	
Maine, USA	Not religious or affiliated with a religion	33%
	Affiliated with a religion	67%
	Christian—60%	
	Other—7%	
The Netherlands	Not religious or affiliated with a religion	42%
	Affiliated with a religion	57%
	Christian—51%	
	Other—6%	
	Religion considered very important	20%
New Jersey, USA	Not religious or affiliated with a religion	19%
	Affiliated with a religion	81%
	Christian—67%	
	Other—14%	
Oregon, USA	Not religious or affiliated with a religion	32%
	Affiliated with a religion	68%
	Christian—61%	
	Other—7%	
Spain	Not religious or affiliated with a religion	19%
	Affiliated with a religion	81%
	Christian—79%	
	Other—2%	
Switzerland	Not religious or affiliated with a religion	12%
	Affiliated with a religion	88%
	Christianity—81%	
	Other—7%	
	Religion considered very important	9%
Vermont, USA	Not religious or affiliated with a religion	38%
	Affiliated with a religion	62%
	Christian—54%	
	Other—8%	
Victoria, Australia	Not religious or affiliated with a religion	32%
	Affiliated with a religion	68%
	Catholic—23%	
	Anglican—9%	
	Other—36%	
	Religion considered very important (Australia-wide)	18%
Washington, USA	Not religious or affiliated with a religion	33%
	Affiliated with a religion	67%
	Christian—61%	
	Other—6%	

Lists countries and states that have some form of legal EPAS with details of their religious make-up. Victorian figures from the Australian Bureau of Statistics 2016. All other data from the Pew Research Center 2015 and 2018

Although understandings of EPAS vary, a widely accepted definition of voluntary euthanasia is the administration of a medication by a health professional to actively end a person's life at the competent individual's voluntary request and with their informed consent (Materstvedt et al., 2003). Physician-assisted suicide is the closely related concept of the prescription of a medication that, when ingested, will result in the person dying (Materstvedt et al., 2003). The more recently introduced terms “voluntary assisted dying” or “medical assistance in dying” describe both active voluntary euthanasia and physician-assisted suicide. Discussions about EPAS are often contextualised to people with terminal medical conditions suffering from pain and distress. Where legal, rules about who can access EPAS vary, ranging from strict criteria related to terminal illness and suffering to those with minimal regulations other than patient autonomy and capacity.

Although not the sole reason, beliefs about the ethics of EPAS are often closely connected to religious beliefs (Sharp, 2018). Many studies have confirmed religiosity to be one of the critical factors associated with people's opposition to the legalisation of EPAS (Chakraborty et al., 2017). A cursory glance at jurisdictions where EPAS has been legalised confirms that these tend towards a greater degree of secularity and atheism than the average (Table 1). Furthermore, in these jurisdictions, among people affiliated with a religion, a lower proportion consider their religious faith important when compared to their counterparts in other parts of the world (Hackett et al., 2018).

Given religious faith appears to play a significant role in defining individuals' beliefs about the ethics of EPAS, it is helpful to understand the official teachings of major religions on EPAS (Eckersley, 2007). The opinions of adherents of any religion may vary significantly between individuals and may sometimes be inconsistent with their religion’s specific official teachings. Likewise, views on the role of pastoral ministry and the beliefs of chaplains and spiritual care workers also vary (Carey et al., 2009; Newell & Carey, 2001). Despite this, a religious organisation's formal theological teachings do influence societal beliefs, often unconsciously, in any given society where that faith predominates (Sandu & Huidu, 2020). A secular Western mindset might presume that the underlying "culture" influences the religious doctrines and beliefs (Eckersley, 2007). However, the reverse is possibly true, i.e. cultural beliefs (both conscious and unconscious) might be influenced by the underlying majority religion (Abdulla, 2018). Therefore, understanding official religious teachings related to life, suffering and death will give significant insight into common and prevailing opinions on the morality of EPAS.

Although atheism is becoming more common in many Western countries, there still tends to remain a majority of citizens who identify as belonging to a particular religion (Hackett et al., 2015). However, in many instances, this affiliation may be nominal without a strong personal connection (Hackett et al., 2018). Outside the West, the situation is even more apparent, with the vast majority of people worldwide identifying with a specific religion. In much of Africa and Asia, religion is playing an increasingly important role in many people’s lives (Hackett et al., 2015).

Of all world religions, four stand out in terms of sheer numbers of adherents: Christianity, Islam, Hinduism and Buddhism (Hackett et al., 2015). Within these four religions, there are then numerous branches or denominations that vary in their specific beliefs and practices.

This article aims to review the official teachings on EPAS of these four major world religions and their principal branches by examining their primary sources of doctrine (with the translated text of the sources quoted in Table 2), using secondary sources to aid this interpretation. Common theological threads that overlap the four religions and influence their teaching on EPAS will then be sought. Finally, the implications of these teachings for ongoing debate and any future implementation of legal EPAS will be explored.

**Table 2:** Primary source [translated] texts that were referred to in the article

Source	Quote
Genesis 1:1 (New Living Translation)	In the beginning God created the heavens and the earth
Colossians 1:17b	He holds all creation together
Matthew 6:27	Can all your worries add a single moment to your life?
Hebrews 1:3	The Son radiates God’s own glory and expresses the very character of God, and he sustains everything by the mighty power of his command
Genesis 1:27	So God created human beings in his own image. In the image of God he created them; male and female he created them
Matthew 6:26	Look at the birds. They don't plant or harvest or store food in barns, for your heavenly Father feeds them. And aren't you far more valuable to him than they are?
Exodus 20:13	You must not murder
Proverbs 24:11–12	Rescue those who are unjustly sentenced to die; save them as they stagger to their death. Don’t excuse yourself y saying, “Look, we didn’t know”. For God understands all hearts, and he sees you
Genesis 9:6	If anyone takes a human life, that person's life will also be taken by human hands. For God made human beings in his own image
Genesis 3:17–19 excerpts	Since you … ate from the tree whose fruit I commanded you not to eat, the ground is cursed because of you. All your life you will struggle to scratch a living from it… until you return to the ground from which you were made. For you were made from dust, and to dust you will return"
Isaiah 66:13	I will comfort you there in Jerusalem as a mother comforts her child
2 Corinthians 1:3b-4a	God is our merciful Father and the source of all comfort. He comforts us in all our troubles so that we can comfort others
Isaiah 53:3	He was despised and rejected, a man of sorrows, acquainted with deepest grief
Revelation 21:4	He will wipe every tear from their eyes, and there will be no more death or sorrow or crying or pain. All these things are gone forever
Ephesians 1:7	He is so rich in kindness and grace that he purchased our freedom with the blood of his Son and forgave our sins
Matthew 5:30	And if your hand – even your stronger hand – causes you to sin, cut it off and throw it away. It is better for you to lose one part of your body than for your whole body to be thrown into hell
Pastoral Constitution Gaudium et Spes, number 27, 1.17	Furthermore, whatever is opposed to life itself, such as any type of murder, genocide, abortion, euthanasia or wilful self-destruction, whatever violates the integrity of the human person… all these things and others of their like are infamies indeed. They poison human society, but they do more harm to those who practice them than those who suffer from the injury. Moreover, they are supreme dishonor to the creator
Sacred Congregation for the Doctrine of the Faith: Declaration of Euthanasia	For it [euthanasia] is a question of the violation of the divine law, an offense against the dignity of the human person, a crime against life, and an attack on humanity
1998 Lambeth Conference, Resolution I.14	This conference … (c) resolves that euthanasia, as precisely defined, is neither compatible with the Christian faith nor should be permitted in civil legislation;
1992 Southern Baptist Convention: Resolution on Euthanasia and Assisted Suicide	Therefore, Be it RESOLVED, That we the messengers to the Southern Baptist Convention, meeting in Indianapolis, Indiana, June 9–11, 1992, affirm the biblical prohibition against the taking of innocent human life by another person, or oneself, through euthanasia or assisted suicide;
2010 AOG General Presbytery in Session: Sanctity of human life: Position Paper	Many factors have energized the right-to-die movement, including sincere concerns over excessive reliance on life-sustaining technologies and inadequate pain-relief care for the terminally ill. Its driving force, however, is a mistaken, deceptive, and evil philosophy that devalues suffering people. Consequently, our opposition to the termination of human life must be understood in spiritual terms and must be guided by biblical principles. Specifically, the Church must (1) proclaim humankind’s dignity as God’s sovereign creation, (2) reassert God’s authority over life from conception to death, and (3) affirm meaning and hope for suffering humanity
John 1:17	For the law was given through Moses, but God’s unfailing love and faithfulness came through Jesus Christ
2 John 1:3	Grace, mercy and peace, which come from God the Father and from Jesus Christ – the Son of the Father – will continue to be with us who live in truth and love
Quran 39:62 (M.A.S. Abdel Haleem Translation)	God is the creator of all things; He has charge of everything
Quran 3:145	No soul may die except with God's permission at a predestined time
Quran 16:61	If God took people to task for the evil they do, He would not leave one living creature on earth, but He reprieves them until an appointed time: when their time comes they cannot delay it for a moment nor can they bring it forward
Quran 17:70	We have honoured the children of Adam and carried them by land and sea; We have provided good sustenance for them and favoured them specially above many of those We have created
Quran 33:72	We offered the Trust to the heavens, the earth, and the mountains, yet they refused to undertake it and were afraid of it; mankind undertook it
Quran 17:33	Do not take life, which God has made sacred, except by right: if anyone is killed wrongfully, We have given authority to the defender of his rights, but he should not be excessive in taking life, for he is already aided [by God]
Quran 4:9	Let those would fear for the future of their own helpless children, if they were to die, show the same concern [for orphans]; let them be mindful of God and speak out for justice
Quran 31:17	Bear anything that happens to you steadfastly
Quran 84:25	But those who believe and do good deeds will have a never-ending reward
Quran 13:24	Peace be with you, because you have remained steadfast. What an excellent reward is this home of yours!
Quran 94:5	So truly where there is hardship there is also ease;
Quran 2:216	Fighting is ordained for you, though you dislike it. You may dislike something although it is good for you, or like something although it is bad for you: God knows and you do not
Sahih Al Bukhari 4.3463 (M. Muhsin Khan Translation)	Allah's Apostle said, "Amongst the nations before you there was a man who got a wound, and growing impatient (with its pain), he took a knife and cut his hand with it and the blood did not stop till he died. Allah said, “My Slave hurried to bring death upon himself so I have forbidden him (to enter) Paradise”
Islamic Organization for Medical Sciences' 1981 Islamic Code of Medical Ethics	Human life is sacred and should not be wilfully taken… A doctor shall not take away life even when motivated by mercy…
Ayatollah Sayyid Ali Khamenei—Islamic Rulings: Medical Issues, Question 115 (Hamid Hussein Waqar Translation)	It is not obligatory to keep the dying person alive or delay his death… But, any action that would cause death would not be permissible
Isha Upanishad verse 3 (Sri Aurobindo Translation)	Sunless are those worlds and enveloped in blind gloom whereto all they in their passing hence resort who are slayers of their souls
Mahabharata 13.117.37–41 (Satguru Sivaya Subramuniyaswami Translation)	Ahimsa is the highest dharma. Ahimsa is the best tapas. Ahimsa is the greatest gift. Ahimsa is the highest self-control. Ahimsa is the highest sacrifice. Ahimsa is the highest power. Ahimsa is the highest friend. Ahimsa is the highest truth. Ahimsa is the highest teaching
Mahatma Gandhi	Should my child be attacked by rabies and there was no hopeful remedy to relieve his agony, I should consider it my duty to take his life
Samyutta Nikaya 56.11 (Bhikku Sujato Translation)	Now this is the noble truth of suffering: birth is suffering, aging is suffering, illness is suffering, death is suffering; union with what is displeasing is suffering; separation from what is pleasing is suffering; not to get what one wants is suffering
Majjhima Nikaya 136.15 and 136.17 (Bhikku Sujato Translation)	I have seen a person here who killed living creatures… And after death, they were reborn in hell… I have seen a person here who refrained from killing living creatures… And I saw that that person was reborn in a heavenly realm
Samyutta Nikaya 45.8	And what is the noble eightfold path? It is right view, right thought, right speech, right action, right livelihood, right effort, right mindfulness, and right immersion And what is right view? Knowing about suffering, the origin of suffering, the cessation of suffering, and the practice that leads to the cessation of suffering. This is called right view
Vinaya Piṭaka, Collection on Monastic Law 3 (Bhikku Brahmali Translation)	On one occasion a certain monk was sick. Feeling compassion, the monks praised death to him. He died. They became remorseful and said, "Could it be that we've committed an offense entailing expulsion?" They told the Master, and he said, "You have committed an offense entailing expulsion"
1948 United Nations Universal Declaration of Human Rights, Article 18	Everyone has the right to freedom of thought, conscience and religion; this right includes freedom to change his religion or belief, and freedom, either alone or in community with others and in public or private, to manifest his religion or belief in teaching, practice, worship and observance

## Methods

A literature review was conducted to identify published secondary texts that examined understandings and interpretations of primary texts of one or more of the branches of the four identified world religions. Articles on Judaism were included in this screening process as the Hebrew scriptures, or Old Testament, is also part of the Christian canon of scripture. Primary texts were defined as both translated ancient holy texts and theological statements published by recognised leaders or official leaderships bodies.

An American Theological Library Association (ATLA) database search for “euthanasia” was performed. Inclusion criteria were: academic publication; written in English; outcome of interest (articles that specifically explored the ethics of euthanasia from the perspective of at least one of the major world religions identified above). One author assessed all abstracts against inclusion criteria. Relevant articles were obtained in full text format. These were assessed for eligibility according to the same criteria and ineligible papers were excluded. The following data were extracted: relevant primary texts and data relevant to the research questions. English translations of each primary text were obtained. Extracted data from primary texts were then analysed primarily using a grammatical–historical approach to exegesis (Surburg, 1974) in addition to understanding them through any lens of the interpretation described in the identified secondary sources.

## Results and Discussion

**Fig. 1:**
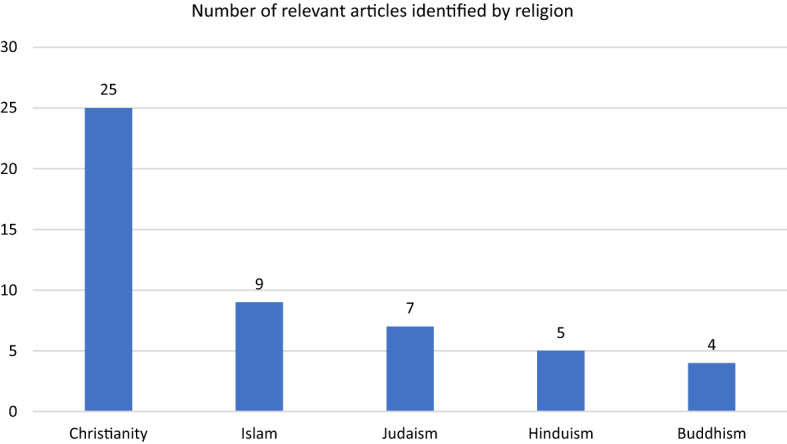
Number of relevant articles identified by religion. Note: Fig. 1 shows the number of articles that explored the ethics of euthanasia from the perspective of at least one of the major world religions identified through an ATLA search for “euthanasia”. Some articles examined more than one religion and are therefore represented more than once in the chart

The ATLA search for “euthanasia” revealed 1,183 articles. Abstract screening identified that 44 articles explored the ethics of euthanasia from the perspective of at least one of the major world religions; a small number of articles examined the topic from the perspective of two or more of these world religions. Publication dates ranged from 1984 to 2020 with half of all articles published within the last 10 years. Twenty-five of the articles related to Christian perspectives on euthanasia (10 specific to Catholicism, 4 to Protestantism and 4 to Orthodoxy), 9 on Islamic viewpoints (3 specific to Shia and 1 to Sunni Islam), 7 on Jewish understandings, 5 on Hindu perspectives and 4 on Buddhist (Fig. 1).

Primary texts identified from these 44 articles included the ancient, scriptural texts of the Bible, the Quran, the Vedic texts and the Pali Canon. Further non-scriptural primary texts identified included *Gaudium et Spes* (The Second Vatican Council, 1965), the Sacred Congregation for the Doctrine of the Faith’s *Declaration on Euthanasia* (1980), resolutions from the Lambeth Conference (1998) and the Southern Baptist Convention (1992), documents from the Assemblies of God General Presbytery in Session (2010), *Sahih Al Bukhari* (ca. 846 C.E./1997), *the Islamic Code of Medical Ethics Kuwait Document* (International Organization for Islamic Medicine, 1981), Ayatollah Sayyid Ali Khamenei’s *Islamic Rulings* (2007) and the *Collected Works of Mahatma Gandhi* (Mahatma Gandhi, 1926).

### Christianity

Christianity is the world’s largest religion in terms of the number of professing adherents (Hackett et al., 2015). It contains various distinct branches that, although divided by historical separations and contemporary leadership structures, mutually recognise each other as part of the worldwide Christian faith. Principal branches include the Catholic Church (and more specifically, Roman Catholicism), Eastern Orthodoxy (which includes the Russian and Greek Orthodox Churches) and the Protestant denominations. Of the many Protestant churches, two distinct themes of Protestantism have emerged in contemporary society, the evangelical, or conservative, Protestant churches and the progressive, or liberal, Protestant churches. In both practice and theology, these two themes are more relevant than denomination when it comes to understanding beliefs (Bauder et al., 2011). For example, an evangelical Anglican church shares closer theological ties with an evangelical Baptist church than it does with a liberal Anglican church.

In this article, the doctrines of the three most populous branches of Christianity worldwide will be analysed: Catholicism, Eastern Orthodoxy and Evangelical Protestantism (which includes many Pentecostal churches). The most important primary source for each of these is the Bible. Other written documents may also be considered primary texts of official church doctrine, although they are less authoritative than the Bible. These include the creedal formulae of church councils in all three branches, and, in the Roman Catholic Church, Papal Encyclicals and the Catechism of the Catholic Church. In more recent decades, denominations have also released specific officially authorised documents about EPAS.

#### Biblical Analysis of Themes that Speak on the Topic of EPAS

Although allusions to euthanasia are found in the literature from the Ancient Near East and the Greco-Roman world (Erdemir & Elcioglu, 2001), the Bible itself is silent on EPAS. However, several themes are spoken about within the Bible that shed light on Christian thinking regarding euthanasia, including creation, human life, suffering and hope.

##### Creation

The Bible views the universe through the monotheistic lens of a creator God who, although able to enter into the universe, exists outside of space–time. From the Old Testament’s first sentence (Genesis 1:1), a picture emerges of a creator who spoke the universe into existence. This creation theme continues throughout the Bible, including into the New Testament, which describes God as the creator of all things (John 1:1–4). Furthermore, although creation within the Bible is spoken of in terms of an event, there is also a theme of ongoing sustaining of the creation by God himself (Colossians 1:17; Hebrews 1:3).

God as the sole creator and sustainer of the universe is an important theological concept, producing a sense of human limitation regarding issues of life and death (Matthew 6:27). The ability of life to be self-sustaining, of the heart to beat and the lungs to breathe, is not merely a natural phenomenon in Biblical terms; rather, this is under God’s control. As such, any human interference with hastening the end of life must be very carefully considered and cautiously approached.

##### Human Value

In the Biblical narrative, humans are seen to be part of the sphere of creation. However, humans have a special place within creation and are especially valuable because they are created in the image of God (Genesis 1:27).

The meaning of “God’s image” is complex and nuanced. However, even without exploring the depth of this, it can be seen, from the Bible’s perspective, that humans are unique. There are many allusions scattered throughout the Bible of this unique value, including from the words of Jesus in the Gospels (Matthew 6:26).

The importance of this view of human value is particularly seen in Biblical passages that describe the consequences of judgement that God anticipates upon those who do not value human life. Direct instructions from God prohibiting murder, mandating its punishment and ensuring justice, occur in several passages such as Exodus 20:13, Genesis 9:6 and Proverbs 24:11–12.

This prohibition on taking life relates intimately to both the value of humans and God’s nature as creator and sustainer. The prohibition against taking life is not absolute, however, as can be seen by the implicit support for capital punishment from Genesis 9:6. Nonetheless, the taking of life is restricted to only distinct circumstances. Furthermore, the framework of God as sustainer indicates that the deliberate action of killing should only ever occur at God’s command. Consequently, there is an implied prohibition of EPAS in Biblical theology. To argue against a Biblical ban on EPAS, it becomes necessary to understand EPAS in one of the two ways. Either EPAS does not involve deliberate killing but is merely facilitating the dying process, or, although EPAS does involve deliberate killing, it is a compassionate representation of love relieving suffering. However, neither argument is strong in overcoming the straightforward scriptural mandate that disallows killing except in the framework of the creator’s direct instruction.

##### Suffering, Death, Hope and Resurrection

Although Genesis’ creation account describes a good creation, the Bible develops a theodicy and theology of suffering very early on. It describes the entrance of suffering into the world through the concept of sin, the act of deliberately turning away from God and his instructions. In the narrative of Genesis 3, the first man and woman make the deliberate choice to ignore God’s instruction to them, and the result is a separation from everlasting life sustained by God (Genesis 3:17–19).

Although suffering and death entered the universe because of sin, the Bible also demonstrates that we are often unable to explain the suffering of an individual. Jesus himself referred to this a number of times in the Gospels (e.g. John 9:2) and the Old Testament book of Job finishes with verses that emphasise the impossibility of humans ever truly understanding the mystery of suffering (Job 42:3).

In offering these two contrasting views of suffering—that it is through human sin that suffering occurs, yet, that suffering cannot be understood fully—the Bible also describes two responses of God to our suffering. Firstly, God cares about human suffering, and secondly, God himself suffers. In many places, the Old Testament describes God’s care for his people Israel and his desire to comfort them in their suffering (e.g. Isaiah 66:13). Likewise, in the New Testament, a picture of Jesus as a comforter emerges. For example, in Matthew 5:4 Jesus is recorded as saying “Blessed are those who mourn for they will be comforted”. The Epistles also share this image of God as a comforter (2 Corinthians 1:3–4).

God is not only portrayed as one who cares and comforts; he is also portrayed as the God who suffers, especially in the person of Jesus, God the Son (Isaiah 53:3).

The suffering that God endured not only places God in a position to understand our suffering but is part of God’s redemptive plan for humanity. The book of Revelation describes the future of humanity as one without suffering. God himself wipes every tear away from his resurrected people who have received eternal life (Revelation 21:4). The redemption is inaugurated in the suffering of Jesus on the Cross (Ephesians 1:7). In other words, God does not delight in human suffering but instead is in the process of bringing suffering and death to an end. At the same time, the Bible acknowledges suffering and death are realities that will be part of the lives of all people in this world.

This Biblical description of suffering and death immersed in hope is complex. Humans, in imitating God’s love and care for his creation, aim to bring relief of suffering and hope to the world. In this sense, this could be extrapolated as Biblical acceptance of EPAS; however, suffering in this life is inevitable according to the Bible, and suffering is not presented as the ultimate of evils as it is transient and temporary. Instead the Bible explains that this life, with its associated suffering and death, occurs along the path to a better future of eternal life without suffering. In this theological framework, living right in this life, following the path set out by God and being obedient to his rules, is of more importance than avoiding temporary suffering (Matthew 5:30). As such, there is a strongly implied theological opposition to EPAS throughout the Bible. Consistent with this, Roman Catholic, Eastern Orthodox and most evangelical Protestant churches present a unified official stance in opposition to EPAS.

#### An Analysis of Roman Catholic Teachings

The Roman Catholic Church has developed official statements and documents consistent with the Biblical analysis on EPAS already described. These documents, including the Second Vatican Council’s *Pastoral Constitution Gaudium et Spes* (1965), the Church’s *Declaration on Euthanasia* (Sacred Congregation for the Doctrine of the Faith, 1980) and Pope John Paul’s *Evangelium Vitae* (1995), explore themes of God as Creator, the intrinsic value of all human life and the sinfulness of deliberately destroying human life. In 1965, the Second Vatican Council made a strong comment on the sanctity of life and expressly condemned euthanasia (*Pastoral Constitution Gaudium et Spes*, Number 27, 1.17).

Catholic doctrine on EPAS is further clarified in its *Declaration on Euthanasia* (Sacred Congregation for the Doctrine of the Faith, 1980), where life is described as a gift from God. Although death is unavoidable, it is the pathway to immortal life. Medical staff are instructed to never neglect their care for the dying and to seek to bring comfort to the ill. Euthanasia, specifically defined as an action undertaken with the express intention of causing death in order to alleviate all suffering, is described as sinful (Sacred Congregation for the Doctrine of the Faith, 1980).

#### An Analysis of Eastern Orthodoxy Teachings

The Orthodox churches have less church-wide official documents that speak about EPAS when compared with Roman Catholicism. However, there is widespread understanding of its leaders of the inherent sinfulness of EPAS. For example, Orthodox priests and, to a lesser extent, parishioners almost universally recognise the Church’s prohibition on euthanasia (Sandu & Huidu, 2020; Verulava et al., 2019) even if they themselves do not specifically agree with it. In standing opposed to the practice of EPAS (Sandu & Huidu, 2020), Eastern Orthodoxy sees a direct link between mercy and salvation as being fundamentally in contradiction with euthanasia (Guroian, 1993). Furthermore, God’s sovereignty is a strong theme of Orthodox theology. As such, an acceptance of illnesses that God has allowed in our lives is an important part of faithful living and opens the Orthodox Christian to spiritual growth (Sandu & Huidu, 2020).

#### An Analysis of Evangelical Protestantism

The Protestant Christian churches present a less unified view on EPAS than do the Catholic and Orthodox churches. Within the evangelical theme of Protestant Christianity, there is a strong emphasis on Biblical authority on matters of faith. These churches therefore naturally tend towards opposing EPAS.

The Anglican Church, one of the largest branches of the Protestant church, although not monolithically evangelical, confirmed a resolution against euthanasia during its 1998 Lambeth Conference of worldwide bishops. This statement affirmed the intrinsic value of all life, explicitly opposing EPAS (Lambeth Conference, Resolution I.14).

Although self-identifying as individually autonomous local churches, Baptist churches have also released numerous statements through their various voluntarily connected associations. For example, the Southern Baptist churches released a *Resolution on Euthanasia and Assisted Suicide*, confirming its belief in the connection between humans created in God’s image and the sacredness of human life being incompatible with EPAS (Southern Baptist Convention, 1992).

The world’s largest association of Pentecostal churches, the Assemblies of God, has also released specific documents that discuss the issue of EPAS and confirm its opposition to the practice. In an analysis of the subject from a Biblical standpoint, the 2010 General Presbytery in Session adopted a *Sanctity on Human Life Position Paper* noting that the philosophy favouring EPAS was “mistaken, deceptive and evil”. In this document, EPAS is described through the lenses of humankind’s dignity, God’s authority and hope for suffering humanity. Despite recognising the sinful nature of EPAS, the document concludes with themes of mercy and grace, as defining elements of the Christian faith (e.g. John 1:17 and 2 John 1:3).

### Islam

Islam, the world’s second-largest religion, has two major branches, Sunni and Shia (Hackett et al., 2015). Both consider the Quran as God’s message and the primary source for determining religious beliefs. In addition to the Quran, Muslims rely on Hadith or records of the words of Islam’s major prophet, Muhammad and Ijma or expert reasoning and consensus (Avci, 2019). Sunni Islam, accounting for almost 80% of Muslims, consists of several distinct schools of interpretation, including Shafi, Hanbali, Hanafi and Maliki. These schools broadly agree on matters of doctrine. Shia Islam, the second-largest branch of Islam, accounting for almost 20% of Muslims, also consists of various similarly minded sub-branches, including the Zaidis, Ismailis and Ithna Asharis.

No Muslim-predominant nations have legalised EPAS. Furthermore, in most Muslim-predominant countries, the degree of secularity and atheism is less than in the West’s traditionally Christian nations, where EPAS has risen to prominence (Hackett & Grim, 2012). Therefore, broader Islamic theological discussions about EPAS have not naturally occurred to the same degree as in Christian denominations. Nonetheless, there has been increasing consideration by contemporary Sunni and Shia scholars of the topic.

#### Quranic Analysis of Themes Related to EPAS

The Quran has minimal direct discussion on the topic of EPAS (Avci, 2019), but it does contain passages about Allah’s authority, human dignity, life, perseverance, death and paradise. These passages give guidance towards an Islamic interpretation that, in general, strongly opposes EPAS in any context (Avci, 2019), considering it to be a form of murder (Sobotkova, 2019).

##### Allah’s Authority

In the Quran, all existence is explained as having come into being through one God, Allah, who is the creator of all life. As the creator, Allah is understood as the trustee over everything in existence (Quran 39:62). He has sole authority over life (Quran 3:145) and death (Quran 16:61), which includes determining the timing of every person’s death. From an Islamic perspective, recognising this truth is an essential part of being a good servant of God (Avci, 2019).

##### Human Value

In addition to Allah’s complete authority over life and death, human life is presented as having special value in Islam and is considered sacred. This is a particular honour given to humanity over all other creatures and is connected with the ability to learn (Quran 17:70) and receive faith (Quran 33:72). Consistent with these axioms of human value and Allah’s authority, murder or the deliberate taking of life outside the context of justice is forbidden (Quran 17:33). In other words, there are certain times when the taking of life is allowable in Islam, but the Quran is explicit that this can occur only when it represents justice for crimes committed or on the instruction of Allah. This prohibition on the taking of life also extends to suicide (Quran 4:29).

##### Suffering, Perseverance, Comfort and Paradise

The Quran’s view of human value extends to a philosophy towards saving life (Sobotkova, 2019). Even in hardships and suffering, there is a command to persevere in Islam (Quran 31:17), which includes patiently bearing through the suffering of illness, either experienced personally or by a loved one.

The need for obedience to Allah’s authority is a central theme in Islam (Avci, 2019). Through choices of obedience to Allah and belief in his instructions, the Muslim travels the road to heaven, or paradise, after a resurrection and day of judgement for all people. In contrast, actions performed in disobedience to Allah and rejection of Quranic teaching result in a judgement of guilt and entry to hell (Quran 84:25). Through obedience to Allah and perseverance in illness, the believing Muslim may have comfort and hope of resurrected life in paradise (Quran 13:24).

Although the Quran speaks particularly of a distant future of resurrection and paradise, it also promises comfort for those suffering in this life. Allah is called gracious and merciful, and comfort is promised in hardship (Quran 94:5). Associated with this is a recognition that a Muslim can, and should, seek spiritual and emotional support to help soften their pain (Avci, 2019). There is also some comfort offered in the Quran’s representation of Allah’s omnipotence with the teaching that Allah understands our hardships but that sometimes these occur for our good (Quran 2:216).

These Quranic descriptions regarding Allah, human life, obedience and paradise weigh against Islamic acceptance of EPAS. Therefore, there is widespread agreement among Islamic scholars and leaders, both Sunni and Shia, that EPAS is in contradiction to Allah’s will (Avci, 2019; Ayuba, 2016; Sobotkova, 2019).

#### An analysis of Sunni Thought

Sunni doctrine and shariah, or law, is strongly guided by the Sunnah and Hadith, in addition to the Quran (Avci, 2019). The *Sahih Al Bukhari*, one of the most trusted Hadith, is explicit in its condemnation of suicide, even when done because of pain (*Sahih Al Bukhari* 3463, ca. 846 C.E./1997).

Throughout history, Islamic scholars have consistently interpreted the Quran and Hadith as prohibiting the act of suicide. From ancient Sunni scholars such as Al-Ghazali in the tenth century to Islamic bodies and statements of the twentieth century such as the *Islamic Medical Code of Ethics* (International Organization for Islamic Medicine, 1981), assisted suicide, and, more recently, euthanasia, remain condemned (Avci, 2019). Contemporary Sunni Islamic advisory and judicial bodies worldwide have issued fatwas, or non-binding legal guidance, prohibiting the practice of EPAS. These bodies include Dar al-Ifta al-Misriyyah in Egypt and the National Fatwa Committee of Malaysia (Malek et al., 2018).

Desire for relief and even praying for death may be acceptable in Islam, however, given Allah’s supremacy over life and death current Sunni thought insists that a higher value be placed on the sanctity of life than on a person’s quality of life (Avci, 2019). Nonetheless, Sunni scholars have recognised distinctions between murder, suicide and assisted suicide. However, all such differences relate to the severity of sin and the degree of punishment required, and EPAS is almost universally considered a form of murder. As such, EPAS remains haram, or prohibited, in Sunni praxis (Ayuba, 2016).

#### An Analysis of Shia Thought

Like Sunni Islam, Shia also determines doctrine by interpreting both the Quran and Hadith. Expert opinion from Shia religious jurists plays a significant role in determining doctrine and practice (Sobotkova, 2019). On bioethical issues, this expert opinion can be arrived at through reason in addition to an exploration of the Quran and Hadith (Dabbagh & Aramesh, 2009). These expert opinions are, at times, pronounced without explanation (Sobotkova, 2019). Although Shia religious jurists’ legal views do vary, the highest-ranking Shia clerics have consistently taught against the practice of EPAS. On this specific question, for example, the Ayatollah Sayyid Ali Khamenei of Iran has confirmed Shia’s prohibition on EPAS (Islamic Rulings: Medical Issues, Question 115, 2007).

### Hinduism

Hinduism, India’s largest and the world’s third-largest religion, does not have a known founder or prophet, nor is there a single, authoritative scripture from which doctrine and belief stem (Avci, 2019). Likewise, no central authorities express conclusive Hindu doctrine. Despite this, strong cultural connections link Hindus, and in practice, the distinction between Indian culture and religion is ambiguous (Avci, 2019). In these shared cultural connections of Hindus are concepts such as atman, shared holy texts such as the Vedas and common rituals (Frazier, 2011). Hinduism is thus a connected yet diverse polytheistic, pantheistic or monistic belief and cultural system containing several loosely defined schools and branches. Sects such as Vaishnavism, Shaivism, Shaktism and Smartism are distinguished through their primary deity. Similarly, schools such as Purva Mimamsa, Yoga and Vedanta are demarcated by their philosophical and scriptural foci. Despite this extensive diversity in Hinduism and lack of centralised authority, the shared cultural, scriptural, ritual and conceptual connections allow for the development of Hindu philosophies for complex ethical issues, including for EPAS. There is less discussion of and uniformity of beliefs on the morality EPAS in Hinduism, however, compared to Christianity, Islam and Buddhism. For example, a recent systematic review on the beliefs regarding EPAS in major world religions did not identify any observational studies on the views of Hindus in the general population (Chakraborty et al., 2017). Furthermore, in the small amount of the published literature about EPAS and Hinduism, it is apparent that Hindu philosophies both against and in favour of EPAS coexist. Thus it is impossible to dogmatically state that Hinduism universally opposes or supports EPAS (Avci, 2019).

#### Hindu Themes Connected with EPAS

Hinduism has a rich history of exploring spiritual issues of atman, life and being, and of death, rebirth and ongoing existence. These are all significant themes that influence Hindu philosophies about EPAS. An additional specific underlying principle of profound importance is that of ahisma, the prohibition of killing.

#### Life and Death

In the Hindu worldview, a person consists of both a mortal physical body and an immortal atman, or soul. In this soul, thoughts and life experiences (Avci, 2019) are distinct from the mortal body. Although death will come to the physical body, the soul will continue its existence after death. Life then does not begin with birth and end with death, but rather exists eternally with the soul reincarnated or reborn into a new body, either human or animal, in a continuous and everlasting cycle of birth, death and rebirth (Ganga, 1994).

#### Dharma and Karma

The cycle of death and rebirth in Hinduism is associated with ideas of balance and justice, although not necessarily in the current life. Dharma is a word that describes the underlying cosmic law and moral force ordering the universe that is maintained through right obligations and behaviour (Avci, 2019; Frazier, 2011). Karma, an associated concept, relates to the moral aspect of a person’s day-to-day conduct (Avci, 2019). If a person lives righteously with good conduct, then there is good karma and a good future in a reborn life is being shaped; likewise, when people live with bad thoughts and actions, they suffer negative consequences in their future.

This idea of balance and justice extends to the circumstances around a person’s death. When a person dies at an old age, in a peaceful manner, in the right place and at the proper time, this is seen as a good death. Conversely, a bad death has occurred when a person dies prematurely, violently, in the wrong place or at the wrong time. Given this, EPAS becomes a problematic practice in Hinduism, with the person dying before the right time (Avci, 2019). However, the issue is not entirely straightforward, as there is some scope for religiously motivated suicide in Hinduism (Ganga, 1994). Historically, from the Hindu viewpoint, this has usually occurred in the context of right intentions and circumstances (Avci, 2019). This potential for religiously motivated suicide opens the door for appropriate EPAS within Hinduism for some; however, more conservative Hindus do not see EPAS as appropriate or desired because suffering is attributed to the law of karma. If one is destined to suffer, then one must fulfil that suffering in this lifetime; otherwise, it will be passed on to the next life (Ganga, 1994). There are allusions to this in various Hindu holy texts (e.g. *Isha Upanishad* verse 3).

#### Ahimsa and Killing

Hinduism has long maintained a philosophy of respect for all living things. It vigorously discourages the act of killing (Avci, 2019). This principle, known as ahimsa, is found in ancient Vedic texts and prayers such as the Riga Veda and Yajurveda (Walli, 1974) and is described as the highest virtue in the Sanskrit epics (*Mahabharata* 13.117.37–41). Ahimsa remains strongly emphasised in Hindu thought and culture today. Mahatma Gandhi, for example, the highly influential Hindu thinker and Indian leader in the last century, was well known for his lifelong commitment of non-violence and ahimsa. This commitment against killing can be understood to speak powerfully against Hindu acceptance of euthanasia (Abbas et al., 2008). However, there is recognition within Hindu thought that, on occasions, a small sin may be committed to avoid greater sins or to demonstrate love (Gielen, 2012). For example, hastening the death through euthanasia of a person suffering intensely from a terminal illness may reflect compassion. Gandhi himself alluded to this at times suggesting that it is not relevant to apply the principle of non-violence to euthanasia where relief of suffering is the goal (Collected Works of Mahatma Gandhi, Volume 37, 1926).

Complex and nuanced threads of thought evidently infuse the Hindu understanding of EPAS. Atman and karma, balance and justice, and non-violence and ahimsa indicate EPAS is not acceptable within Hindu thought. However, an absolute exclusion of EPAS cannot fairly be made as Hinduism does allow scope for religiously motivated suicide and, possibly, for compassionately motivated EPAS. Despite this, contemporary Indian culture and its legal system is clear in its rejection of EPAS (Sinha & Sarkhel, 2012).

### Buddhism

Almost 7% of the world’s people, primarily in Southern and Eastern Asia, identify as Buddhist, a religion founded by Siddhartha Gautama, also known as the Buddha. Texts believed to be records of his oral teachings, especially the Pali Canon collection, play a central role in Buddhist doctrine and are generally considered authoritative (Keown, 1998). Other religious texts are also often recognised as scripture, although it is difficult to define the limits of what constitutes scripture as views differ between the branches of Buddhism. Furthermore, there is no authoritative Buddhist council that speaks on behalf of the religion (Keown, 1998). Buddhism has also developed into several schools or branches with some differences in both practice and interpretation of the Buddha’s teachings. Significant branches include Theravada, Mahayana and Vajrayana, although there are many smaller branches.

Given the lack of a universally agreed corpus of scripture and absent contemporary central authority, along with the numerous branches of Buddhism, it is difficult to articulate a definitive Buddhist view on EPAS. However, specific themes within Buddhism provide a framework for understanding EPAS from a Buddhist perspective. These themes include concepts related to life, suffering, moral living and karma, death, rebirth and enlightenment.

#### Life and Suffering

Buddhist doctrine is built on the Four Noble Truths, which describe a philosophy of suffering. These truths are described in the Pali Canon and various other scriptures. The first of these truths contends that suffering is an innate aspect of life (*Samyutta Nikaya* 56.11).

Buddhism presents suffering as an undeniable reality, explaining in the second noble truth that misplaced desire, ignorance and hatred are the root causes of all suffering. There is, however, potential for liberation of suffering in Buddhist philosophy and the way to discover the end of suffering is described in the third and fourth noble truths. The principle behind ending suffering, the third noble truth, involves removing all desires, and this is achieved through the eightfold path illustrated in the fourth noble truth (Keown, 1998). Thus, Buddhism has a highly developed philosophy of the reality and cause of suffering. The way to reach enlightenment and exist in a state without suffering is the central underlying premise permeating all Buddhist thought.

#### Karma and Rebirth

As in Hinduism, the ideas of karma’s balance and justice, and cyclical existence through rebirth are dominant themes within Buddhism. Karma is seen as an inherent part of the natural order (Keown, 2000) where intentional actions, thoughts and words influence future conditions in a reborn life. In other words, right and moral thoughts and actions, including preserving life, lead, at some point in time, to good rebirths (*Majjhima Nikaya* 136.15). Likewise, wrong and immoral thoughts and behaviours, including killing, at some point in time, result in unhappy rebirths (*Majjhima Nikaya* 136.17).

Karma is central to the Buddhist worldview and has a significant logical influence on the understanding of EPAS. As suffering is part of the universe’s natural order of balance and justice, EPAS is unable to alleviate suffering and will merely delay it to a subsequent life. The ending of a life is not the solution to suffering because life is seen through the prism of this cyclical existence of death and rebirth. And in fact, the desire for death sits squarely at odds with the Buddhist philosophy that calls for all desires to be extinguished (Keown, 1998). Rather than through death, Buddhism teaches that suffering is overcome through enlightenment which is reached through following the eightfold path of righteousness. This eightfold path includes cultivating a moral and right view, right intentions and right conduct, right speech, right conduct, a right livelihood, right effort, right mindfulness and right concentration. Through following the eightfold path, death and suffering cease, and liberation is attained (*Samyutta Nikaya* 45.8). This path includes living morally through right action, which extends to avoiding killing, suicide and assisting suicide. Buddhist scriptures specifically tackle the question of assisted suicide (*Vinaya Piṭaka*, Collection on Monastic Law), denying its rightness (Kawanda, 2016). Likewise, suicide is considered wrong except in the case of the Buddhist who has finally removed all desires and has completed their work and is ready to pass to Nirvana (Keown, 1998).

Buddhism aims for its adherents to find liberation from suffering, but not through death. In line with this, Buddhist teaching seeks to comfort those suffering with illness, helping them find peace that transcends their suffering.

### Common Themes

Of particular interest is the observation that all four of the major world religions paint EPAS in a negative light. Their holy texts, scholars and official bodies consistently speak against the moral acceptability of EPAS. This contrasts with a secular and atheistic worldview where EPAS is frequently supported (DeCesare, 2000). The locus of morality of all four religions may play a role in this contrast. A secular or atheist worldview might contend that morality comes from within as an inherent, internal characteristic of each individual. Alternatively, this worldview may assume the need for a consequentialist ethic where outcome defines an action’s morality. In contrast, all four religions describe an external arbiter of morality in the form of deontological philosophy. In Christianity and Islam, this authority is God as revealed in either the Bible or Quran, and in Hinduism and Buddhism, the arbiter is the universal cosmic force or dharma. This inevitably leads practitioners of these religions to seek to understand morality not from within but instead in accordance with this external arbiter and as shown, all four religions develop a strong deontologically based position that it is inherently wrong to intentionally kill another human.

A second overlapping theme in these religions is the doctrine of life continuing after death. All four religions contend that life continues after death, either a resurrected life in heaven or hell in Christianity and Islam or a reborn life in this world in Hinduism and Buddhism. This ongoing existence may be good and with less or no suffering. Alternatively, it also may be worse with continued suffering. The afterlife state in which a person finds themselves is intimately related to the person’s actions in their previous life. Thus, the actions a person takes in this life have a very real consequence for their next life, so they must carefully consider all their deeds. As all four religions prohibit the taking of life and consider killing sinful, it follows that the hope of heaven or a better rebirth will lead to an antipathy to any action that intends to end a life. In other words, advocating for EPAS, even in the face of suffering, exposes a person to the risk of negative consequences in their next life.

### Implications

The fundamental worldviews of the world’s four major religions are against the moral acceptability of EPAS. Not only do these beliefs represent thousands of years of combined human wisdom, but also the views of billions of people living today. There will thus be ongoing concern regarding the implementation of EPAS. Although this will be especially apparent in countries where religion officially plays a more direct role in government, it is also likely to be observed in any country with a high proportion of the population who identify as religious. Given this, lawmakers should ensure the views of those in leadership roles in the major religions remain part of any discussion regarding EPAS. The wide range of views on EPAS is recognised by governments in that politicians are usually allowed a conscience vote by their parties when it comes to EPAS legislation.

There is a need to genuinely consider the issue of conscientious objection, both at an individual level and at an institutional level, in jurisdictions that make a decision to legalise EPAS. At the individual level, clinicians who oppose the practice of EPAS, including many who adhere to one of these four religions, may experience distress if they feel compelled, by law or otherwise, to defy the teachings of their religion and take part in any aspect of the process of EPAS (Stevens, 2006). From a pragmatic perspective, safeguards against coercion to provide EPAS will reduce the risk of the loss of these clinicians who might otherwise resign from clinical practice, thereby depriving like-minded patients of access to the services of practitioners who share their moral views as well as being detrimental to the individuals concerned (Quinlan, 2016). At a more fundamental level, governments that legalise EPAS without ensuring provisions for clinicians to opt out will be violating the basic human right of clinicians to exercise their freedom of religious belief and practice as outlined in the *Universal Declaration of Human Rights* (United Nations, 1948).

“Everyone has the right to freedom of thought, conscience and religion; this right includes freedom to change his religion or belief, and freedom, either alone or in community with others and in public or private, to manifest his religion or belief in teaching, practice, worship and observance”.

Universal Declaration of Human Rights, Article 18

In much of the world, hospitals, aged care facilities, healthcare services and health insurance providers are, to some degree, privately operated, often by religious organisations. For example, the Roman Catholic Church is the largest non-government worldwide provider of healthcare services (Agnew, 2010). As is the case for individual conscientious objection, legislating bodies should consider the issue of institutional conscientious objection. The idea of institutional conscientious objection is controversial given that institutions are not autonomous individuals and therefore, arguably, they lack the capacity to have a conscience (Bedford, 2016). Furthermore, when institutional conscientious objection is codified, there are potential occasions where individual clinicians are compelled to disregard the instructions of their own conscience in determining medical care in deference to institutional policies (Spencer, 2011), although prospective employees are made aware of institutional requirements and are asked to comply prior to commencing employment. It can also be argued that an institution relies on social agency where its actions occur through its members. This requires “socially coordinated behavior to enable each component part to pursue the institutional ends in harmony” (Bedford, 2016). Regardless of the theoretical position on institutional conscience taken, significant practical ramifications can be imagined in jurisdictions that legalise EPAS without allowing for institutional “opt outs”. These ramifications might include animosity between government and religious institutions, an impression of persecution of religious institutions by government, the collapse of private healthcare providers with subsequent economic and healthcare consequences and an increased burden on public healthcare systems. The existence of institutions not involved in EPAS would also allow like-minded patients to choose a healthcare provider that aligned to their values—such patients would not find themselves involved in unwanted discussions about EPAS with their clinicians. In this sense, removing provisions of conscientious objection would actually reduce rather than increase patient choice, which is often the purported goal of EPAS legislation.

Some commentators have suggested that the move to legalise EPAS, after it has been prohibited for millennia, despite a capacity to relieve pain better than ever before, is not due to a change in the situations of individuals who seek EPAS, but instead indicates profound changes in contemporary postmodern, secular, Western societies (Sommerville, 1996). These include the growth of individualism and the desire for autonomy, and the decline of religiosity with its vocabulary that allows for discussion around death and tolerance of mystery. Further research is required to explore whether religion might be a protective measure against annihilation of the self and the role of religion in generating hope in this context.

## Conclusions

A review of the primary sources of the world’s four major religions demonstrates a near-universal opposition to the legalisation and practice of EPAS due to themes revolving around a common hope for a better life after death and a system of morality that is fundamentally deontological. Despite this opposition, there is increased discussion of and momentum towards state-sanctioned EPAS, particularly in Europe, North America and Australasia. Given these facts, it is especially important for legislators to explore and understand key religious viewpoints when considering legalising EPAS and enshrine the capacity for conscientious objection at an institutional and individual level.

## Data Availability

Not applicable.
